# Effects of a wholegrain-rich diet on markers of colonic fermentation and bowel function and their associations with the gut microbiome: a randomised controlled cross-over trial

**DOI:** 10.3389/fnut.2023.1187165

**Published:** 2023-06-01

**Authors:** Nicola Procházková, Naomi Venlet, Mathias L. Hansen, Christian B. Lieberoth, Lars Ove Dragsted, Martin I. Bahl, Tine Rask Licht, Michiel Kleerebezem, Lotte Lauritzen, Henrik M. Roager

**Affiliations:** ^1^Department of Nutrition, Exercise and Sports, University of Copenhagen, Frederiksberg, Denmark; ^2^Host-Microbe Interactomics, Wageningen University and Research, Wageningen, Netherlands; ^3^National Food Institute, Technical University of Denmark, Kgs. Lyngby, Denmark

**Keywords:** energy harvest, dietary fibres, whole-grain diet, refined-grain diet, gut microbiota, colonic transit time

## Abstract

**Background:**

Diets rich in whole grains are associated with health benefits. Yet, it remains unclear whether the benefits are mediated by changes in gut function and fermentation.

**Objective:**

We explored the effects of whole-grain vs. refined-grain diets on markers of colonic fermentation and bowel function, as well as their associations with the gut microbiome.

**Methods:**

Fifty overweight individuals with increased metabolic risk and a high habitual intake of whole grains (~69 g/day) completed a randomised cross-over trial with two 8-week dietary intervention periods comprising a whole-grain diet (≥75 g/day) and a refined-grain diet (<10 g/day), separated by a washout period of ≥6  weeks. A range of markers of colonic fermentation and bowel function were assessed before and after each intervention.

**Results:**

The whole-grain diet increased the levels of faecal butyrate (*p* = 0.015) and caproate (*p* = 0.013) compared to the refined-grain diet. No changes in other faecal SCFA, BCFA or urinary levels of microbial-derived proteolytic markers between the two interventions were observed. Similarly, faecal pH remained unchanged. Faecal pH did however increase (*p* = 0.030) after the refined-grain diet compared to the baseline. Stool frequency was lower at the end of the refined-grain period compared to the end of the whole-grain diet (*p* = 0.001). No difference in faecal water content was observed between the intervention periods, however, faecal water content increased following the whole-grain period compared to the baseline (*p* = 0.007). Dry stool energy density was unaffected by the dietary interventions. Nevertheless, it explained 4.7% of the gut microbiome variation at the end of the refined-grain diet, while faecal pH and colonic transit time explained 4.3 and 5%, respectively. Several butyrate-producers (e.g., *Faecalibacterium, Roseburia, Butyriciococcus*) were inversely associated with colonic transit time and/or faecal pH, while the mucin-degraders *Akkermansia* and Ruminococcaceae showed the opposite association.

**Conclusion:**

Compared with the refined-grain diet, the whole-grain diet increased faecal butyrate and caproate concentrations as well as stool frequency, emphasising that differences between whole and refined grains affect both colonic fermentation and bowel habits.

## 1. Introduction

Diet is one of the key factors influencing gut microbiota composition and metabolism (1, 2). In particular, dietary fibres and whole grains, that are rich in fibres, are poorly digested by the host, serving as a major substrate for colonic fermentation by the residential microbes (3). In return, the gut microbiota produces short-chain fatty acids (SCFA), which are known to exert beneficial effects on the host (4). Moreover, dietary fibre is an important regulator of bowel function including stool output, water content, and intestinal transit time, some of which are attributed to its water-binding properties (5). We previously reported that a whole grain diet decreased body weight and serum inflammatory markers as compared to a refined-grain diet, emphasising its health benefits (6). Suggested mechanisms behind the inverse relationship between dietary fibres and body weight include greater stool outputs and higher total energy excretion in the stools (7, 8). Intake of dietary fibres appears to decrease the digestibility and absorption of fats and proteins, thereby reducing the energy available to the host (8, 9). *In vivo* studies suggest that some of these effects are mediated by the gut microbiota since the microbial community influences the amount of energy extracted from the diet (10–12). Further supporting this hypothesis, a recent human study found that decreased caloric intake is associated with widespread changes to the microbiome composition and limited microbiome nutrient harvesting capacity, resulting in increased stool energy excretion (13). Stool energy density has additionally been reported to be associated with intestinal transit time and microbiome community structures (14). A higher intake of whole grains might increase the microbial production of SCFA (15, 16) which among many other functions act as signalling molecules contributing to whole-body energy homeostasis (17) and exert anti-inflammatory effects (18). Furthermore, diets rich in fibres have been associated with lower levels of metabolites resulting from microbial proteolysis, some of which are implicated in disease (19, 20). On the contrary, refined grains are produced from whole grains by processing (e.g., milling or heating), which ultimately leads to the deprivation of their nutritional value and fibre content (21). Some scientific evidence suggests an increased risk of metabolic syndrome or higher blood lipids associated with consumption of refined grains but with inconsistent results (22, 23) and the effects of refined grains on the colonic environment is limited. Here, we explored the effects of whole-grain (WG) vs. refined-grain (RG) diets on markers of colonic fermentation and bowel function, as well as their associations with the gut microbiome.

## 2. Materials and methods

### 2.1. Study design

As described in Roager et al., 50 subjects (18 men and 32 women) exhibiting an increased metabolic risk completed a randomised, controlled cross-over study with two 8-week dietary intervention periods comprising a whole-grain diet and a refined grain diet in random order, separated by a wash-out period of at least 6 weeks (6). The whole-grain consumption was ≥75 g/day and < 10 g/day during the whole-grain period and the refined-grain period, respectively. Participants were advised to replace all cereal products from their diet with the provided study products, corresponding to the intervention. A total of four examination days, before and after each intervention, were conducted. At visits 1, 2, and 4, a 4-day pre-coded dietary registration developed by the National Food Institute at the Technical University of Denmark was assessed to determine the total dietary intake of whole grains, total energy and macronutrient intake (*n* = 150) (24). Diet adherence was assessed by reviewing a study diary, which was maintained by the participants, as well as by measuring fasting plasma alkylresorcinols, which are biomarkers of whole grain intake (25). On each examination visit, participants arrived in the morning after having fasted for ≥10 h (h) and abstained from strenuous physical activity for ≥10 h and alcohol consumption for ≥24 h. Stool samples were collected in the morning of each examination day and were stored at 5°C for maximally 24 h (*n* = 200). The stool samples were homogenized in sterile water 1:1 on a weight based volume and stored at −80°C. A total of 195 faecal samples were retrieved from the freezer for this study (visit 1, *n* = 49; visit 2, *n* = 49; visit 3, *n* = 48; visit 4, *n* = 49). Before examination days at visits 1, 2, and 4, participants ingested non-absorbable radio-opaque transit markers for six consecutive days, which allowed the estimation of colonic transit time (CTT, *n* = 150) as previously described (6, 26). In addition, participants recorded the number of defecations per day in a defecation diary during these six consecutive days. Based on these records, the stool frequency (average bowel movements per day) was calculated for each participant at the first baseline (visit 1) and after each intervention (visits 2 and 4), respectively. The study was conducted at the Department of Nutrition, Exercise and Sports at the University of Copenhagen (Denmark) between July 2012 and November 2013. The study received approval from the Municipal Ethical Committee of the Capital Region of Denmark in accordance with the Helsinki declaration (H-2-2012-065) and the Data Protection Agency (2007-54-0269) and was registered at www.clinicaltrials.gov (NCT01731366).

### 2.2. Gut microbiota characterization

Microbial DNA was extracted from faecal samples according to the ‘MetaHIT’ method, as described previously (27, 28). Characterization of the gut microbiota was performed by 16S rRNA gene sequencing as previously reported (6, 26).

### 2.3. Faecal pH, water, and energy density

The homogenized faecal samples, which were stored at −80°C, were thawed overnight. The wet aliquots were weighed and thoroughly mixed by a vortex. Faecal pH was measured with a pH meter (PH-208) and the pH meter was cleaned with distilled water between every pH measurement. Next, the lids of the cryotubes, containing 1 mL of homogenized material, were removed and the material was dried at 50°C in an oven for 72 h. After drying, the total dry weight was measured. The dry sample weight ranged from 0.02–0.1 g, depending on the total wet weight. All weighing measurements were done using AG204 Delta Range scale with an accuracy of 0.1 mg. The percentage of faecal water content was then calculated. The stool energy density (J/g) of the dried faeces was obtained by heat combustion with the use of a bomb calorimeter (IKA C6000). A benzoic acid tablet (C723 IKA) was used for calibration and was added to each measurement to ensure ignition.

### 2.4. Urine metabolomics and faecal short-chain fatty acids

The urine metabolomes were obtained as previously published (6). In brief, the urine samples were profiled by liquid chromatography-mass spectrometry (LC–MS) and metabolites of microbial proteolysis (p-cresol sulfate, p-cresol glucuronide, indoxyl sulfate and phenylacetylglutamine) were identified. SCFA were quantified in faecal samples by targeted UPLC-QTOF-MS (Waters) in negative ionization mode as previously published (29). In brief, homogenized faecal samples were derivatized using 3-nitrophenylhydrazine (3NPH). Derivatized ^13^C_6_-SCFA-analogues (acetic acid, propionic acid, butyric acid, isobutyric acid, 2-methylbutyric acid, isovaleric acid, valeric acid, caproic acid, 3-methylvaleric acid and isocaproic acid, respectively) were produced and used as isotope-labelled internal standards. The raw LC–MS data were analysed using QuanLynx (Waters Corporation). The calibration curves were established by plotting the peak area ratios between the individual SCFA analytes and labelled internal SCFA standards against the concentrations of the calibration standards. The calibration curves were fitted to linear regression. The average *R*^2^ of all external standard calibration curves was 0.98.

### 2.5. Statistical analysis

Dry stool energy density and faecal water content data were checked for outliers after inspection of the corresponding QQ plots. In total, nine measurements of the dry stool energy density were excluded from the subsequent analyses due to failed measurements or technical outliers (*n* = 186). Moreover, four outliers of the faecal water content, with values ranging from 7–30%, were also excluded (*n* = 191). Descriptive and univariate statistical analyses were performed in GraphPad Prism (version 9.4.1). Model assumptions on normality were assessed by visual inspection of residual plots and by D’Agostino & Pearson test (*p* > 0.05). Values were reported as means ±SD unless otherwise noted. The log2 fold changes from the baseline in whole-grain and refined-grain intervention groups were calculated by dividing the intervention endpoint values by the baseline values and subsequent log2 transformation. For the pairwise comparisons between the baselines and the intervention endpoints, and the comparisons between the fold changes, a paired *t*-test or a Wilcoxon paired test was used depending on the distribution of the data. *p*-value below 0.05 was considered statistically significant. Exploratory association analyses were conducted by Spearman’s correlation test at the intervention endpoints and the *p*-values were adjusted for multiple testing by the Benjamini–Hochberg procedure (*q*-value) (30). A *q*-value below 0.05 was considered statistically significant.

Distance-based redundancy analysis (db-RDA) was performed to find variables significantly contributing to the inter-individual variation in gut microbiota composition. The analysis was performed at the genus level with Bray–Curtis dissimilarity using the *capscale* function as implemented in the *vegan* package (version 2.6) in R (version 4.2). The multivariate model selection was performed using the *ordiR2step* function with forward selection. The statistical significance was determined by permutation test and a value of *p* < 0.05 was considered significant. Species associated with the effector variables were determined by extracting the RDA scores (Supplementary Table S1).

## 3. Results

A total of 50 subjects, aged 48.6 years ±11.1, completed the two dietary intervention periods with whole-grain consumption of 69 ± 46 g/day (mean ± SD) at baseline; 179 ± 50 g/day after the whole-grain diet; and 12 ± 10 g/day after the refined grain diet as previously reported (6). The intake of dietary fibre was following: 23 ± 9 g/day at baseline, 33 ± 10 g/day after the whole grain diet; and 21 ± 7 g/day after the refined-grain diet. Compliance was confirmed by both food diaries and plasma alkylresorcinols levels. The intake of nutrients and food groups did not differ between the two diets, except for the intake of dietary fibre and whole grains, as previously reported (6).

### 3.1. Effects of intervention diets on markers of colonic fermentation and bowel function

To investigate the effects of the whole-grain diet and the refined diet on colonic fermentation, we assessed changes in faecal SCFA, faecal BCFA, faecal pH, urinary markers of microbial proteolysis, dry stool energy density, faecal water content, and stool consistency and frequency before and after each intervention.

**Figure 1:**
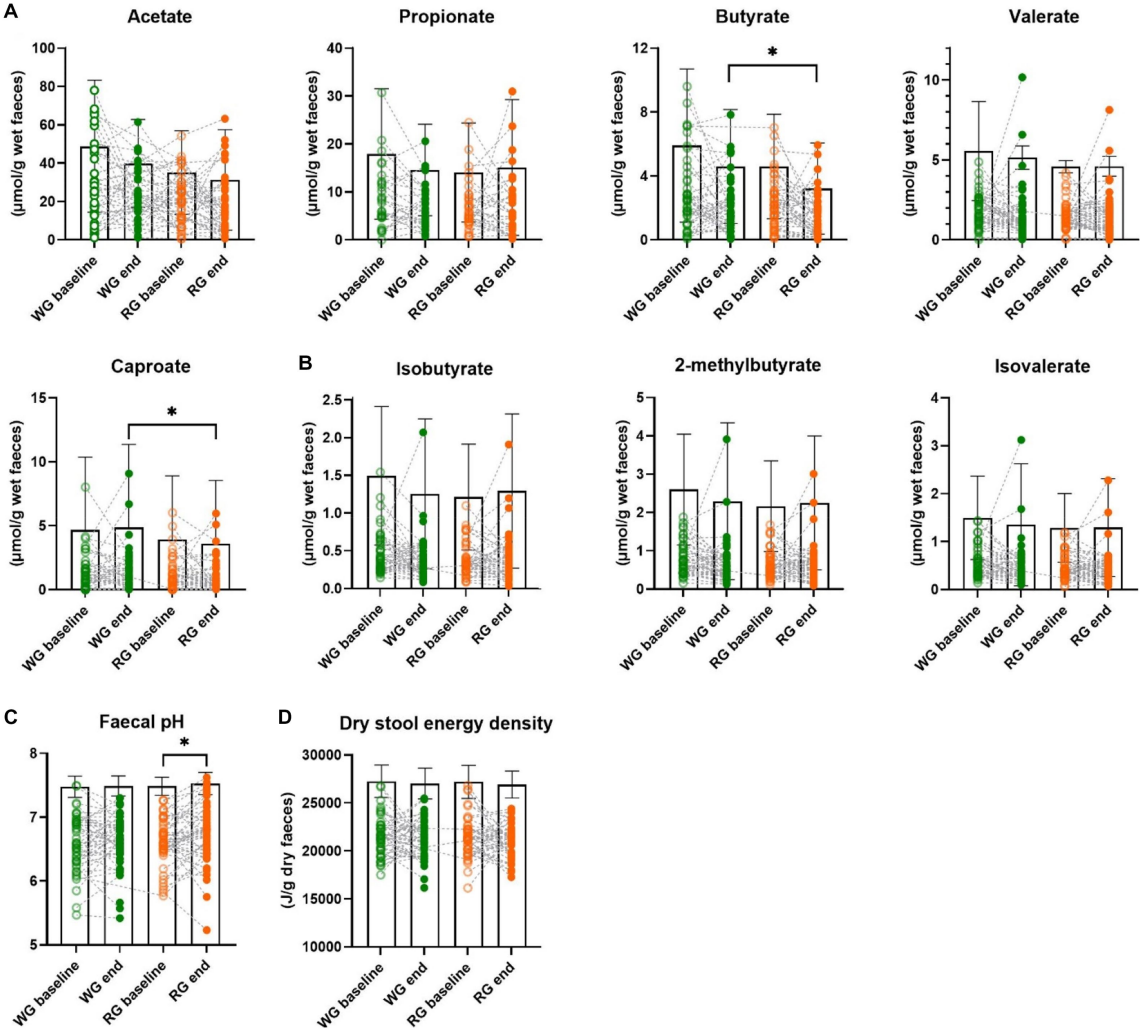
Effects of the intervention diets on markers of colonic fermentation. **(A)** Faecal short-chain fatty acids concentrations, **(B)** faecal branched-chain fatty acids concentrations, **(C)** faecal pH, and **(D)** dry stool energy density before and after the whole-grain diet and refined diet, respectively. Each data point represents an individual, column bars indicate group means and error bars the standard deviation. Empty circles represent baseline samples while the filled circles represent samples after the interventions, green – whole-grain intervention, orange – refined-grain intervention. Asterisks represent significant differences between two-time points (*p < 0.05, Wilcoxon paired test). WG, wholegrain; RG, refined grain.

Higher faecal levels of butyrate were observed at the end of the whole-grain diet in comparison to the refined diet (μmol/g wet faeces mean ± SD, WG: 2.27 ± 1.78 RG: 1.58 ± 1.43; Wilcoxon paired test, *p* = 0.015). The same was observed for caproate (WG: 1.28 ± 1.74 RG: 0.94 ± 1.32; Wilcoxon paired test, *p* = 0.013) whereas no changes in other faecal SCFA nor BCFA were observed between the two interventions (Figures 1A,B; Supplementary Figure S1A). We did find that the percentage of faecal butyrate was significantly higher at the end of the whole-grain diet in comparison to the refined diet (WG: 10.33 ± 6.78 RG: 6.87 ± 5.83; Wilcoxon paired test, *p* = 0.0002; Supplementary Figure S1B) and similarly, the fold change was significantly higher in the whole-grain group in comparison to the refined group (WG: 0.12 ± 1.05 RG: −0.50 ± 1.43; Wilcoxon paired test, *p* = 0.0049; Supplementary Figure S2A). Moreover, faecal pH was unchanged between the two intervention groups. However, faecal pH significantly increased following the refined-grain diet as compared to the baseline (pH baseline: 6.62 ± 0.38 RG: 6.73 ± 0.46; Wilcoxon paired test, *p* = 0.013; Figure 1C), suggesting a decline in the saccharolytic fermentation as reflected by the lower faecal levels of butyrate in the refined-grain group. In addition, the intervention study did not result in any differential changes in dry stool energy density (J/g dry faeces WG: 21240 ± 2024 RG: 21114 ± 1760, Figure 1D).

**Figure 2:**
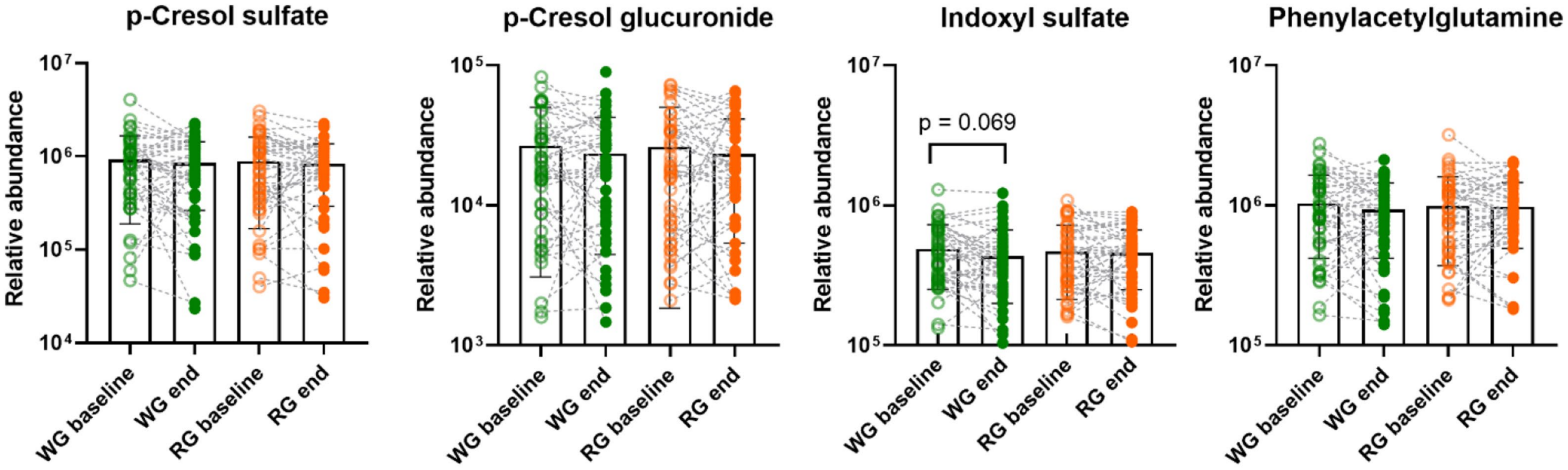
Effects of the intervention diets on urinary markers of microbial proteolysis. Each data point represents an individual, column bars indicate group means and error bars the standard deviation. Empty circles represent baseline samples while the filled circles represent samples after the interventions, green – whole-grain intervention, orange – refined-grain intervention. WG, wholegrain; RG, refined grain.

No differences in urinary markers of microbial proteolysis including p-cresol sulfate, p-cresol glucuronide, and phenylacetylglutamine were observed between the dietary interventions (Figure 2). However, indoxyl sulfate tended (*p* = 0.069) to decrease at the end of the whole-grain intervention compared to the baseline (log relative abundance baseline: 5.64 ± 0.21 WG: 5.57 ± 0.23) and a negative correlation between indoxyl sulfate and dietary fibres (rho = −0.17, *p* = 0.05, Supplementary Figure S3A) was found. Notably, no correlations between intake of dietary fibre and the other proteolytic metabolites were observed (Supplementary Figure S3A).

**Figure 3:**
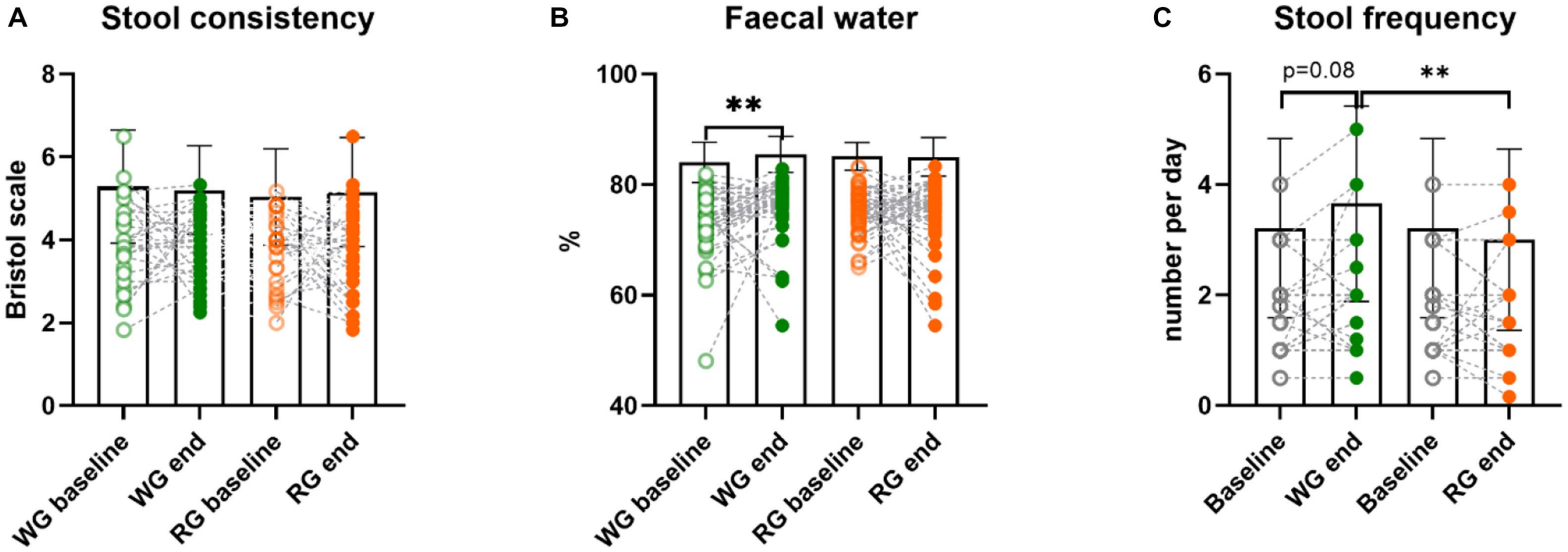
Effects of the intervention diets on markers of bowel function. **(A)** stool consistency assessed by the Bristol stool score (higher score indicates looser stools), **(B)** faecal water, and **(C)** stool frequency (average bowel movements per day), before and after the whole-grain diet and refined diet, respectively. Stool frequency was only assessed at visits 1, 2, and 4, which is why the same baseline appears twice in the graph. Each data point represents an individual (in case of stool frequency the individuals substantially overlap), column bars indicate group means and error bars the standard deviation. Empty circles represent baseline samples while the filled circles represent samples after the interventions, green – whole-grain intervention, orange – refined-grain intervention. Asterisks represent significant differences between two-time points (**p < 0.01, Wilcoxon paired test or paired *t*-test). WG, wholegrain; RG, refined grain.

We previously reported (6) that neither stool consistency assessed by the Bristol stool score (BSS, Figure 3A) nor CTT were significantly altered by the two interventions. Nonetheless, here we found that the faecal water content, a more objective measure of stool consistency, increased after the whole-grain intervention compared to baseline (% baseline: 73.4 ± 6.0 WG: 75.9 ± 5.4; Wilcoxon paired test, *p* = 0.007; Figure 3B) but not between the two intervention periods. A tendency for a positive correlation between the faecal water content and intake of dietary fibre was observed at the end of the whole-grain intervention (rho = 0.26, *p* = 0.08) but not at the end of the refined-grain diet (Supplementary Figure S3B). Furthermore, stool frequency was also significantly higher at the end of the whole-grain intervention compared to the end of the refined-grain intervention (bowel movements/day median (25th, 75th percentile) WG: 2 (1, 2) RG: 1 (1, 2); Wilcoxon paired test, *p* = 0.001) and tended to be increased compared to the baseline (baseline: 1 (1, 2); Wilcoxon paired test, *p* = 0.08; Figure 3C). Altogether, these results indicate the reduction in intake of whole grains during the refined-grain intervention decreased the saccharolytic fermentation in the colon, whereas the increase of whole grains during the whole-grain intervention mainly changed the bowel habits.

### 3.2. Associations between markers of colonic fermentation and bowel function

**Figure 4:**
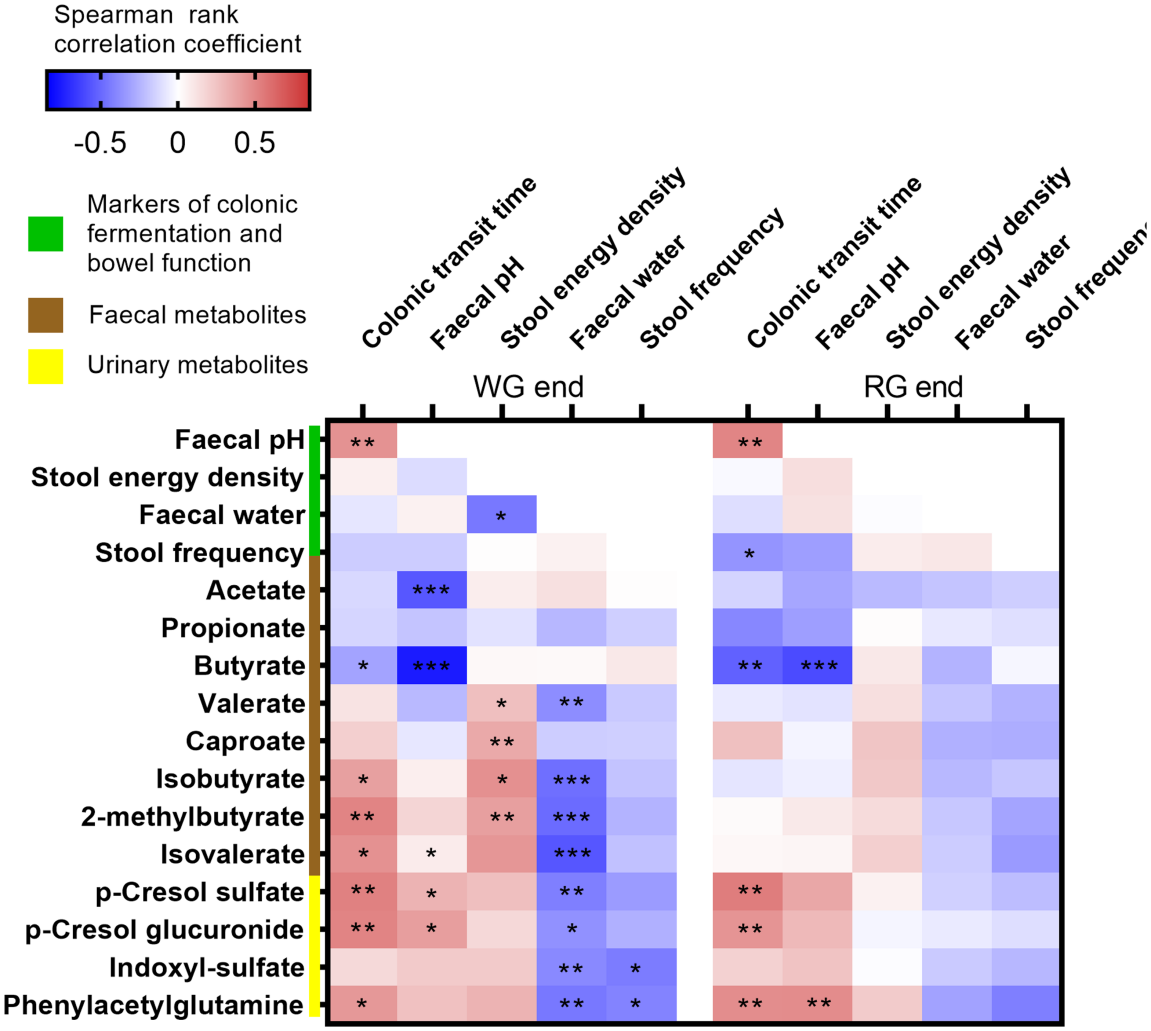
Correlations between markers of colonic fermentation and bowel habits. Heatmap showing Spearman’s rank correlations between the measures of colonic fermentation (i.e., faecal pH, stool energy density, faecal short-chain fatty acids, branched-chain fatty acids, proteolysis markers) and bowel function (i.e., faecal water, stool frequency, and colonic transit time) at the end of the two interventions. Significant associations are represented by asterisks (*q < 0.05, **q < 0.01, ***q < 0.001). WG, whole grain; RG, refined grain.

We next explored associations between the markers of colonic fermentation and bowel function including CTT at the end of the two intervention periods. In line with previously reported human studies (31), faecal pH was strongly inversely associated with faecal butyrate at both end points and with faecal acetate at the end of the whole-grain diet (Figure 4). Longer CTT was consistently associated with higher faecal pH and similarly, we found an inverse correlation between CTT and faecal butyrate at the end of both interventions. Interestingly, longer CTT, lower faecal water content, and greater dry stool energy density were associated with higher levels of faecal BCFA (isobutyrate, 2-methylbutyrate, and isovalerate) at the end of the whole-grain intervention with the same trend observed at the end of the refined diet. BCFA are produced by the gut microbiota from branched-chain amino acids thus reflecting microbial proteolysis and have been previously associated with longer CTT in healthy adults (32). In agreement, proteolytic markers measured in urine including p-cresol sulfate, p-cresol glucuronide, indoxyl sulfate, and phenylacetylglutamine were positively associated with CTT and faecal pH at both time points emphasising that these markers are strongly linked to transit time and colonic fermentation.

### 3.3. Links between markers of colonic fermentation and the gut microbiome composition

Assuming that the changes in colonic fermentation following the refined diet would be reflected in the gut microbiome structure, we investigated how the markers of colonic fermentation and transit time related to the gut microbiome composition using the distance-based redundancy analysis (db-RDA).

**Figure 5:**
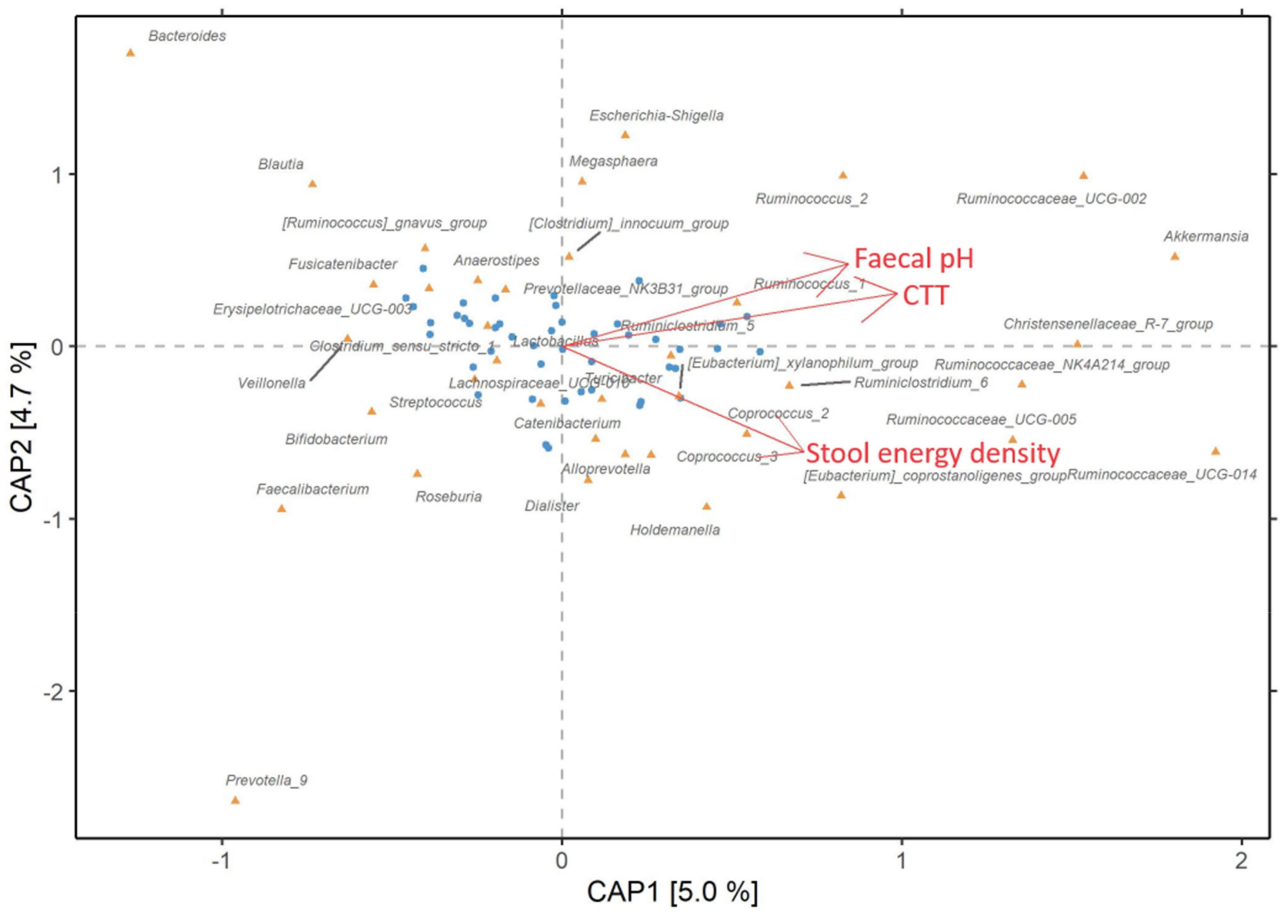
Links between markers of colonic fermentation and gut microbiota composition. Distance-based redundancy analysis (db-RDA) of the gut microbiota composition at genus level with Bray–Curtis dissimilarity at the end of the refined-grain intervention (*n* = 45) constrained by CTT, dry stool energy density, and faecal pH. The blue dots represent the study participants while the orange triangles show bacterial genera associated with the constrained variables. The axes CAP1 and CAP2 are analogous to principal components 1 and 2 and the brackets show explained variance, respectively. CTT, colonic transit time; CAP, constrained analysis of principal coordinates.

The analysis showed that CTT, dry stool energy density, and faecal pH were significantly associated with the gut microbiome composition and explained individually 5, 4.7, and 4.3% of the microbiome variation, respectively (*p* = 0.012, *p* = 0.020, and *p* = 0.035, respectively). To determine which set of variables resulted in the most statistically significant model, we used an automatic model selection and found that CTT and dry stool energy density (*p* = 0.02, *p* = 0.01, respectively) but not pH contributed to the inter-individual variation in the gut microbiome at genus level and cumulatively explained 9% of the variation. Since CTT and faecal pH were highly correlated at the end of the refined-grain diet (rho = 0.51, *p* < 0.001, Figure 4), they likely explain similar trends in gut microbiome variation. When examining associations between bacterial taxa and these markers (Figure 5 and Supplementary Table S1), we noticed that several bacterial genera of the Rumminococaceae family were positively associated with dry stool energy density as previously reported (14). On the contrary, the abundant *Bacteroides* genus was negatively associated with dry stool energy density, in agreement with our recent findings, where the *Bacteroides* enterotype was linked to lower dry stool energy density and higher BMI (14). Rumminococaceae and *Akkermansia* were both positively associated with faecal pH and CTT, whereas several rather abundant fibre-degrading bacterial taxa including *Blautia, Faecalibacterium, Aanaerostipes*, and *Roseburia*, were negatively associated with CTT, and others such as *Butyriciococcus* and *Prevotella* also showed negative associations to faecal pH. It thus appears that with increasing CTT and faecal pH, the relative abundance of fibre-degrading bacterial taxa decreases and the abundance of mucin-degraders such as *Akkermancia* (33) and Rumminococaceae (34) increases.

## 4. Discussion

Whole-grain-rich diets are associated with health benefits including improved blood lipids, reduced inflammatory markers, and weight loss as compared to diets high in refined grains (6, 35, 36). Some of these benefits might be mediated by changes in gut function and fermentation by the gut microbiota. Here, we investigated the effects of whole-grain and refined-grain diets on markers of colonic fermentation and bowel function in a randomised controlled cross-over trial.

We found moderate effects of the whole-grain diet on colonic fermentation reflected by higher faecal concentrations of butyrate and caproate in the whole-grain group compared to the refined-grain group. This may suggest higher SCFA production after the increased intake of whole grains as higher plasma concentrations of butyrate after the whole-grain intervention compared to the refined-grain intervention were reported in our previous study based on this RCT (6).

Despite the increase in faecal butyrate and caproate and increased consumption of dietary fibres on the whole-grain diet, no differences were observed in the faecal pH or other SCFA between the two interventions. It has previously been reported that a combination of resistant starch and wheat bran significantly reduced faecal pH while wheat bran alone did not (37). Similarly, another RCT showed that whole-grain barley did significantly reduce faecal pH and increase butyrate and total SCFA when compared to refined cereal foods whereas no such changes were observed for whole-grain wheat (38). Since dietary fibres vary in their fermentability by the gut microbiota (e.g., inulin is highly fermentable and psyllium is poorly fermentable), the lack of reduction of faecal pH on the whole-grain diet in our study might result from insufficient amounts of fermentable fibres and/or by low intake of slowly fermententable fibres that can reach the distal colon. On the other hand, faecal pH was increased at the end of the refined-grain diet when compared to the baseline. Since faecal pH is largely driven by the presence of SCFA and other organic acids derived from colonic metabolism of the microbiota, the reduction in the fermentable substrate on the refined diet may explain the increase in faecal pH. Nonetheless, a few studies showed that whole grain consumption increases plasma and faecal levels of ferulic acid and other phenolics, which could potentially influence colonic pH (39, 40), however, none of the studies assessed faecal pH. Furthermore, since our study included Danish individuals with a rather high habitual intake of whole grains, the change to the refined-grain diet represented a more substantial dietary change compared to the change to the whole-grain diet.

We observed a tendency for a decrease in urinary levels of indoxyl sulfate, a uremic toxin resulting from the microbial conversion of tryptophan into indole (41), at the end of the whole-grain diet as compared to the baseline, which was correlated to dietary fibre intake. Dietary fibre has previously been associated with decreased circulating serum levels of indoxyl sulfate in patients with chronic kidney disease (19). However, we did not observe any changes in other proteolytic metabolites including faecal BCFA and urinary p-cresol sulfate, p-cresol glucuronide, or phenylacetylglutamine, suggesting that the 10 g increase in dietary fibres during the whole-grain period did not influence the microbial protein catabolism. An RCT with haemodialysis patients showed a significant reduction in serum indoxyl sulfate but not in p-cresol sulfate following a 6-week diet supplemented with resistant starch (16 g/day) (20). Furthermore, another trial with haemodialysis patients showed that supplementation with oligofructose-enriched inulin (10–20 g) for 4 weeks resulted in reduced levels of serum p-cresol sulfate, but not indoxyl sulfate (42), together suggesting that mixtures of specific fibre types might be necessary for decreasing several markers of microbial proteolysis simultaneously.

We did not observe any changes in dry stool energy density with either of the interventions compared to baseline, similar to a previous parallel-arm controlled-feeding trial with whole grains (43), suggesting that the dry stool energy density is rather person-specific and to some extent independent of diet. Consistent with previous studies (44–46), the whole-grain group had increased stool frequency compared to the refined-grain diet. Dietary fibre increases faecal water content through water-holding capacities and faecal bulking, which can lead to more frequent bowel movements (44, 47, 48). Therefore, the increase in stool frequency during the whole-grain intervention was likely mediated by the increased dietary fibre intake during that period. In support, the whole-grain diet increased faecal water content compared to baseline and there was a tendency for a positive correlation between the faecal water content and dietary fibre at the end of the whole-grain intervention. Despite the lack of total faecal excretion measurement in the present study, the fact that the whole-grain diet increased faecal water content suggests an increased bulking effect and a possible increase in total faecal output volume.

As previously reported (49), longer CTT was associated with higher faecal pH, and lower faecal butyrate during both interventions indicating that transit time and colonic fermentation are interrelated independently of the diet. Moreover, longer CTT, lower faecal water content, and higher dry stool energy density were associated with higher BCFA levels in faeces in accordance with a study in healthy participants (32). Unlike SCFA, BCFA originates from microbial proteolysis, suggesting that longer passage throughout the colon might change substrate availability and/or the microbiome structure and its activity (50). Similarly, the protein-derived metabolites p-cresol sulfate, p-cresol glucuronide, indoxyl sulfate, and phenylacetylglutamine in urine were positively associated with CTT, faecal pH, and negatively associated with faecal water content as previously reported (26). Counterintuitively, we observed an inverse relationship between dry stool energy density and faecal water content at the end of the whole-grain intervention. As lower faecal water content reflects longer CTT, this observation might be attributed to the fact that individuals with longer CTT harbour a gut microbiome that is linked to higher dry stool energy density, suggesting a less efficient microbiome-dependent energy extraction consistent with our recent findings (14). Our results thus indicate that not only diet but also bowel function influence colonic fermentation.

BSS has been previously found to explain 4% of inter-individual variation in the gut microbiome (51), here we found that CTT, dry stool energy density, and faecal pH, significantly contributed to the inter-individual variation in the gut microbiome structure explaining 5, 4.7, and 4.3%, respectively. While several genera from the Rumminococaceae family were associated with greater dry stool energy density, the opposite was true for *Bacteroides*. Indeed, the *Bacteroides* enterotype has been recently linked to lower dry stool energy density and higher BMI, whereas the *Rumminococaceae* enterotype showed an inverse relationship (14), supporting the hypothesis that the colonic energy harvest is dependent on gut microbiota composition. As enterotypes have been associated with long-term dietary habits (1) and were reported to be stable during a 6-months dietary intervention (52), the 8-week dietary intervention applied here might not be sufficiently long to modulate colonic energy harvest.

Butyrate-producing species consistently show an inverse relationship with long transit time and/or chronic constipation (50, 53, 54). In agreement herewith, we found a negative association between the relative abundance of *Faecalibacterium, Roseburia*, *Butyriciococcus,* and CTT. Walker et al. found that high colonic pH, here associated with longer CTT, lowers butyrate production and decreases the abundance of butyrate-producing bacteria (55). However, the colonic pH is largely affected by the presence of SCFA and other organic acids, therefore, whether the decrease in butyrate producers observed with high faecal pH is a consequence or a cause needs further investigation.

Strengths of this study include its cross-over design, allowing us to investigate the effects of whole-grain and refined-grain diets on markers of colonic fermentation and bowel function with minimum confounders. Moreover, the measurement of plasma alkylresorcinols, established biomarkers of wholegrain consumption, allowed for monitoring of adherence to the intervention diets (6). The study population included overweight individuals with an increased metabolic risk profile and with a high habitual intake of whole grains, thus findings of this study cannot necessarily be extrapolated to other populations.

In conclusion, we found larger effects of the refined-grain diet on the colonic fermentation compared with the whole-grain diet including an increase in faecal pH. The increase in faecal pH was negatively associated with many butyrate-producing bacteria, emphasising the importance of whole grains as substrates for saccharolytic fermentation. On the contrary, the whole-grain diet moderately affected bowel function including increased faecal water content, which was negatively associated with several metabolites originating from microbial proteolysis. Thus, increasing colonic water content and/or decreasing transit time via intake of dietary fibres and whole grains might be beneficial in attenuating microbial proteolysis implicated in disease. Finally, although there were no differences in dry stool energy density between the whole-grain and refined-grain diets, dry stool energy density was associated with gut microbiota composition, indicative of an interplay between energy harvest and microbiome community composition. Further studies are warranted to disentangle the interactions between specific fibres, gut microbiota, and energy extraction from the diet.

## Data availability statement

The datasets presented in this study can be found in online repositories. The names of the repository/repositories and accession number(s) can be found in the article/Supplementary material.

## Ethics statement

The studies involving human participants were reviewed and approved by the Municipal Ethical Committee of the Capital Region of Denmark (H-2-2012-065). The patients/participants provided their written informed consent to participate in this study.

## Author contributions

MB, MK, and HR conceived the study. NP and NV performed the data analyses. NV measured faecal pH, faecal water content, and stool energy density. NP, MH, and CL performed the quantification of SCFA in faeces under supervision of LD and HR. LL and TL designed and initiated the human study. NP, NV, and HR wrote the manuscript. All authors contributed to the article and approved the submitted version.

## Funding

This study was supported by the Novo Nordisk Foundation (NNF19OC0056246; PRIMA—toward Personalized dietary Recommendations based on the Interaction between diet, Microbiome and Abiotic conditions in the gut).

## Conflict of interest

The authors declare that the research was conducted in the absence of any commercial or financial relationships that could be construed as a potential conflict of interest.

## Publisher’s note

All claims expressed in this article are solely those of the authors and do not necessarily represent those of their affiliated organizations, or those of the publisher, the editors and the reviewers. Any product that may be evaluated in this article, or claim that may be made by its manufacturer, is not guaranteed or endorsed by the publisher.
