# Supplementary material for: Effects of a wholegrain-rich diet on markers of colonic fermentation and bowel function and their associations with the gut microbiome: a randomised controlled cross-over trial
Source: Front Nutr. 2023 Jun 1;10:1187165. doi: 10.3389/fnut.2023.1187165 (PMC10267323; doi:10.3389/fnut.2023.1187165)
Supplement: Supplementary file 2 [file Data_Sheet_1.PDF]

Supplementary Material

Effects of a wholegrain-rich diet on markers of colonic fermentation and bowel function and their associations to the gut microbiome: a randomised controlled cross-over trial

Nicola Procházková†, Naomi Venlet†, Mathias L. Hansen, Christian B. Lieberoth, Lars Ove Dragsted, Martin I. Bahl, Tine Rask Licht, Michiel Kleerebezem, Lotte Lauritzen, Henrik M. Roager*

† These authors contributed equally to the work

*** Correspondence:** Corresponding Author: hero@nexs.ku.dk

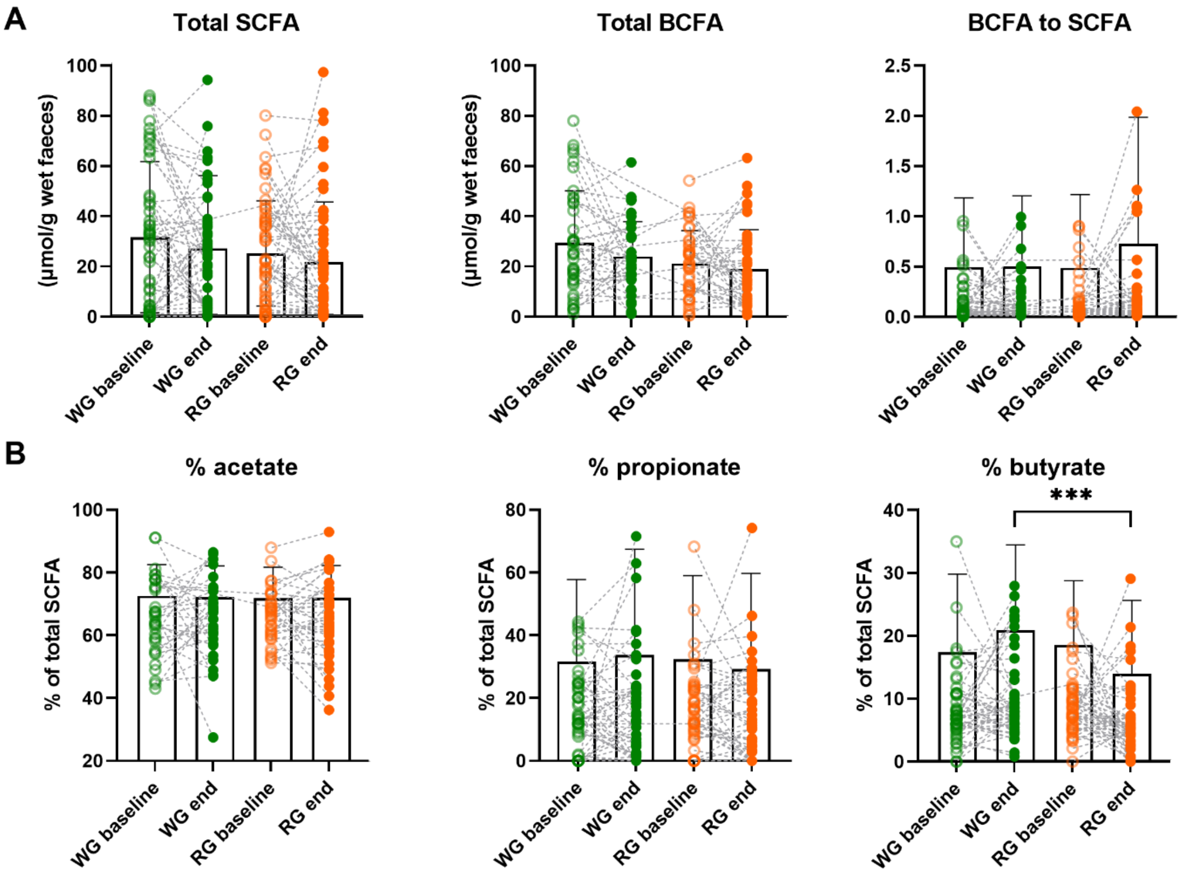

Supplementary Figure 1. Effects of the intervention diets on markers of colonic fermentation. (A) Total faecal short-chain fatty acids (SCFA), branched-chain fatty acids (BCFA) concentrations, and BCFA to SCFA ratio, respectively (B) percentage of acetate, propionate, and butyrate of the total SCFA, respectively. Each data point represents an individual, column bars indicate group means and error bars the standard deviation. Empty circles represent baseline samples while the filled circles represent samples after the interventions, green – whole-grain intervention, orange – refined-grain intervention. Asterisks represent significant differences between two-time points (***$p < 0.001$, Wilcoxon paired test). WG, wholegrain; RG, refined grain.

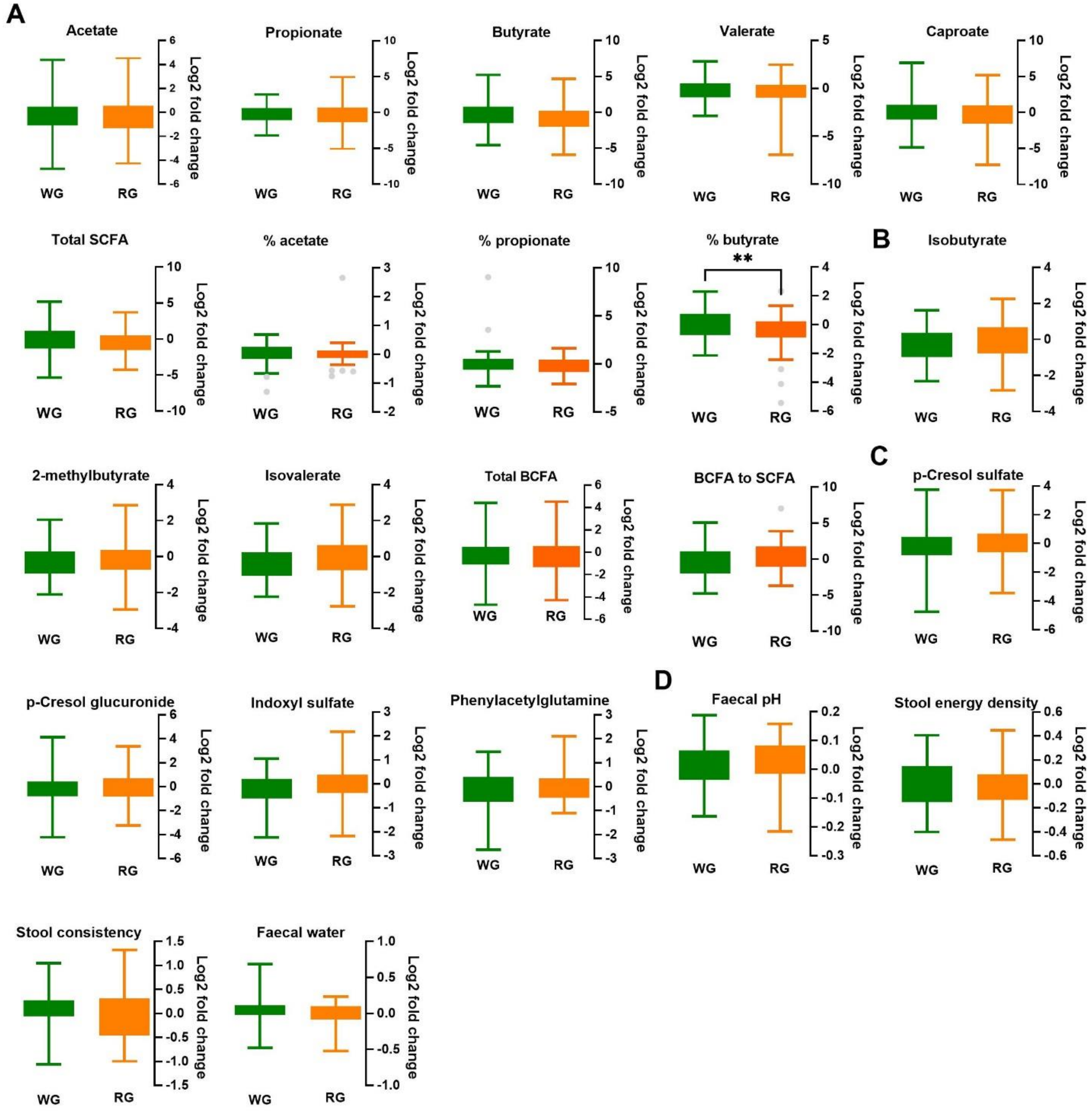

Supplementary Figure 2. Fold changes for (A) faecal short-chain fatty acids (SCFA), (B) branched-chain fatty acids (BCFA), (C) urinary proteolytic markers, (D) other markers of colonic fermentation and bowel habits during the two dietary interventions. The fold changes are shown as boxplots with Tukey whiskers and were calculated by dividing the intervention endpoints values by the baseline values and log2 transformed. Asterisks represent significant differences between the two interventions (**p < 0.01, Wilcoxon paired test). WG, wholegrain; RG, refined grain.

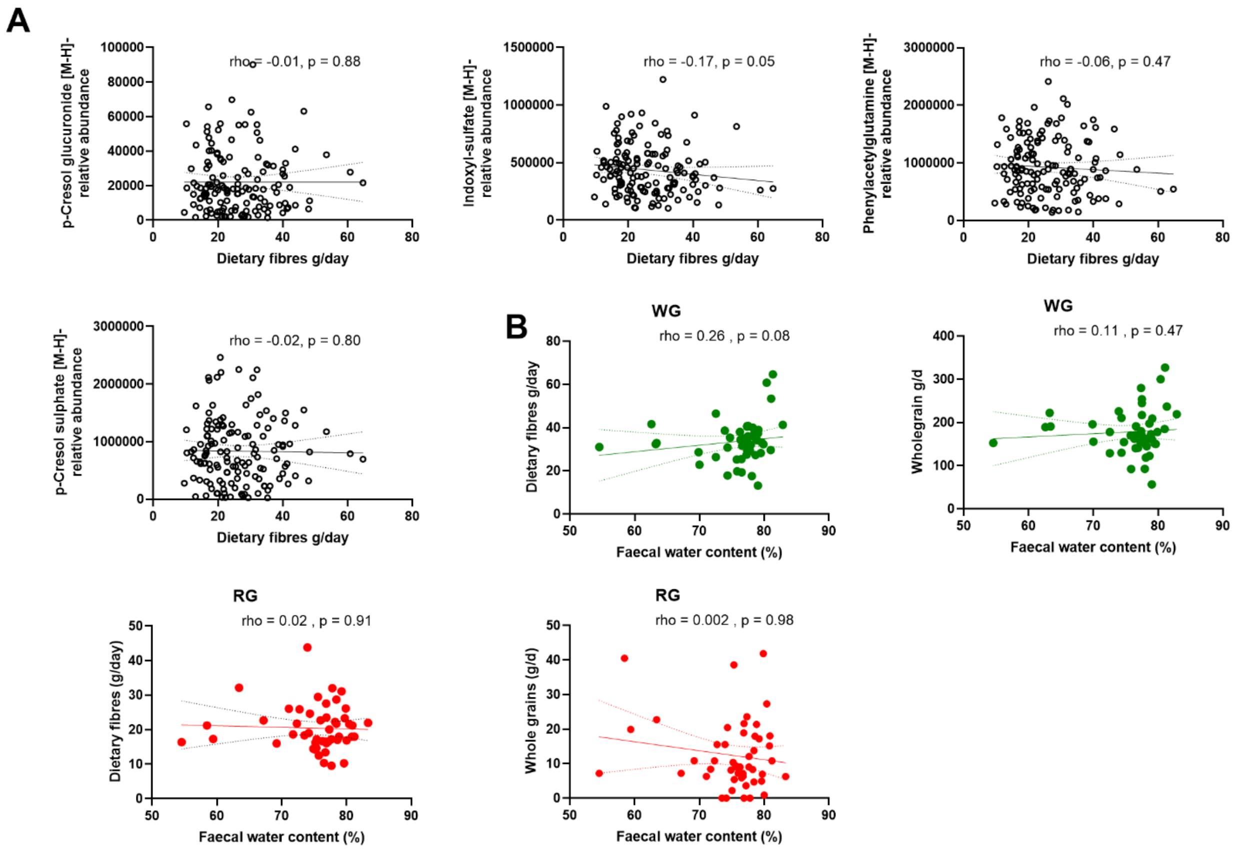

Supplementary Figure 3. (A) Spearman Rank correlations between habitual dietary fibres intake and proteolytic markers measured in urine, (B) Spearman Rank correlations between faecal water content and dietary fibre, and whole grain intake after the whole-grain (green) and refined-grain (orange) interventions, respectively. WG, wholegrain; RG, refined grain.
